# Danggui Buxue Decoction Ameliorates Inflammatory Bowel Disease by Improving Inflammation and Rebuilding Intestinal Mucosal Barrier

**DOI:** 10.1155/2021/8853141

**Published:** 2021-01-19

**Authors:** Chengyin Li, Fenglin Zhu, Shasha Wang, Jing Wang, Bin Wu

**Affiliations:** ^1^Department of Rheumatology, Chongqing Traditional Chinese Medicine Hospital, Chongqing 400021, China; ^2^No. 4 Clinical Medicine School, Chengdu University of Traditional Chinese Medicine, Chongqing 400021, China

## Abstract

**Objective:**

This study aimed to determine whether Danggui Buxue decoction (DGBX) can improve inflammatory bowel disease (IBD) by regulating immunity and promoting intestinal mucosal repair.

**Method:**

Dextran sulfate sodium (DSS) was used to induce the IBD model. Drugs (DGBX or saline) were administered to mice, which were randomly divided into three groups (control, model, and experimental groups). Hematoxylin and eosin staining of intestinal tissues was conducted to observe for morphological changes. Changes in cytokines and immune cells in the intestinal tissues were detected by enzyme-linked immunosorbent assay and flow cytometry. Immunofluorescence techniques were used to assess the status of the intestinal mucosal repair.

**Results:**

This study found that treatment with DGBX can effectively improve the inflammatory state and pathological structure of the IBD model. DGBX not only can significantly change the composition of intestinal mucosal immune cells and promote the regression of inflammation but also significantly increase the proliferation of intestinal epithelial cells and promote the rapid repair of intestinal mucosal barrier injury compared with the model group (*p* < 0.05).

**Conclusion:**

Taking these results, DGBX shows promising protective effects on IBD by regulating immunity and promoting intestinal mucosal repair.

## 1. Introduction

Inflammatory bowel disease (IBD), including ulcerative colitis and Crohn's disease, is characterized by chronic, recurrent gastrointestinal inflammation [1]. IBD is a global health problem, and its prevalence is rapidly rising in all populations. The risk of colorectal cancer in IBD patients is 2 to 8 times that of non-IBD patients [2, 3]. Drug treatment for IBD has thus far failed to achieve its optimal effect: aminosalicylate exhibits moderate efficacy; steroids are effective but can lead to serious complications; biotherapy such as the antitumor necrosis factor-*α* antibody is linked to an increase in opportunistic infection in some patients [4]. Therefore, new safe and effective treatments need to be developed.

During the onset of IBD, the imbalance of intestinal mucosal immunity and the destruction of the intestinal epithelial barrier function play an important role [5, 6]. Various immune cell populations, including macrophages, dendritic cells (DCs), neutrophils, and eosinophils in intestinal mucosa may aggravate inflammation during IBD [7]. These types of cells initiate inflammation by secreting chemokines and proinflammatory cytokines, including IL-6 and IL-1*β*. Regulatory T cells (Tregs) limit IBD inflammation via immunosuppression; thus, the metastasis of Tregs can eliminate the development of experimental colitis [8]. Bone marrow-derived suppressor cells (MDSCs) exert an immunosuppressive effect on both innate and adaptive immune systems to promote immune tolerance [9]. Similarly, adoptive transfer of MDSCs can improve colitis in different animal models of IBD [10, 11]. Intestinal epithelial cells comprise the primary barrier that protects the body. The imbalance of the intestinal mucosal immunity results in a large number of inflammatory factors such as IL-1*β*, TNF-a, IL-6, IL-10, and IL-17A. This process causes damage to the intestinal epithelial cells and the destruction of the intestinal barrier function, ultimately leading to the occurrence and development of IBD [12, 13].

Traditional Chinese medicine, also called complementary and alternative medicine, has been used for thousands of years. Danggui Buxue decoction (DGBX) is a representative prescription of traditional Chinese medicine for stimulating blood production [14]. It is composed of *Angelica sinensis* and *Astragalus membranaceus* in a mass ratio of 1 : 5. DGBX has been used to treat blood-deficiency diseases in the clinical setting for nearly 800 years. Many studies suggest that the role of DGBX in promoting hematopoiesis is related to its ability to regulate immune abnormalities. DGBX can regulate the differentiation of T lymphocytes, resulting in immunosuppressive and hematogenous functions in an immune-induced aplastic anemia mouse model [15–17]. Ferulic acid, the main component of DGBX, enhances hematopoietic cell activity and myeloid cells (including MDSC, DCs, and macrophages) development by the upregulation of granulocyte-colony stimulating factor (G-CSF) and erythropoietin production [18]. Our early experiments also confirmed that DGBX can reverse the differentiation of erythroid progenitor cells, which weakens the immune response to tumor cells [19]. In cultured T-lymphocytes, DGBX markedly induced cell proliferation, secretion of interleukin-2, and phosphorylation of extracellular signal-regulated kinase [20]. Thus, DGBX exerts the effects of regulating the immune status both in vitro and in vivo. Therefore, DGBX has been used in the treatment of immune-related diseases, such as pulmonary fibrosis, atopic dermatitis, and asthma [21–24]. Regardless, the ability of DGBX to improve IBD by regulating immunity or promoting repair is largely unknown.

Our goal for this study is to identify the effects of DGBX on IBD and its mechanism. Considering the remarkable effect of DGBX on immune status regulation, we speculate that DGBX may have a protective effect on IBD. Here, we found that DGBX could significantly improve the clinical symptoms, inflammatory state, and pathological structure of the dextran sulfate sodium- (DSS-) induced IBD mouse models. The underlying molecular mechanisms demonstrated that DGBX not only significantly changes the composition of intestinal mucosal immune cells in IBD but also significantly improves the proliferation and repair of intestinal epithelial cells. These results provide a reliable experimental basis for the potential clinical application of DGBX in the treatment of IBD.

## 2. Methods

### 2.1. Preparation and Quality Control of DGBX

Radix Astragali and *Angelica sinensis* were supplied by Tongrentang Company (Chongqing, China). The morphological identification of plant materials was completed by Dr Xu at the Pharmacy Department, Chongqing Traditional Chinese Medicine Hospital. DGBX was prepared by decocting the crude drugs Astragali Radix and *Angelica sinensis* Radix in a weight ratio of 5 : 1. The decoction was concentrated to 1.5 g/mL of the crude drugs. In accordance with the Pharmacopoeia of the People's Republic of China, ferulic acid was selected as the quality control substance. The concentration of ferulic acid was determined by high-performance liquid chromatography-mass spectrometry. The ferulic acid reference substance was dissolved in 50% methanol to 500 mg/mL and further diluted into different concentrations of the reference substance solution. The experimental methods and results were reported in our previous study [19].

### 2.2. Animals and Treatment

Specific pathogen-free C57BL/6J female mice aged 8 weeks were purchased from Huafukang Biotechnology Co., Ltd. (Beijing, China). The mice were housed and maintained in laminar flow cabinets under specific pathogen-free conditions. DSS was used to build a model of enteritis, which is similar to human IBD in symptoms and pathology. A mouse model was induced with 2.5% DSS in drinking water for 7 d and given normal drinking water for the remaining days [9]. On day 9, the mice were killed and analyzed. A 2.5% DSS reagent was purchased from MP Biomedicals Company (California, USA). The mice in the experimental group were administered intragastrically with DGBX (30 g/kg daily) on day 0 for the prevention model or on day 4 for the treatment model (more than half of the mice developed symptoms), until the ninth day. The dose given was based on our preliminary experiments. Meanwhile, the control group received normal saline. The body weight and clinical symptoms of the mice were recorded daily. At the end of the experiments, the mice were anesthetized with 1% pentobarbital sodium and sacrificed by cervical dislocation. The colon length was recorded, and the colon tissue sections were stained with hematoxylin and eosin (HE). They were subsequently evaluated by histopathological scoring. All experimental procedures were approved by the Institutional Animal Care and Use Committee (IACUC), Chongqing Traditional Chinese Medicine Hospital, in accordance with the approved guidelines set forth by IACUC.

### 2.3. Detection by Enzyme-Linked Immunosorbent Assay

The mice were killed on day 9 after DSS was induced, the colonic tissue of the mice was removed and washed with phosphate-buffered saline (PBS), and the tissue was cut into pieces. The tissue was precooled with PBS and homogenized on ice at 1 : 9. The tissue liquid was poured into the centrifuge tube and then centrifuged at 4°C at 5000 g for 5 min. The supernatant was removed and then stored at −20°C. The protein concentrations of IL-1*β*, TNF-*α*, IL-6, IL-10, and IL-17A were detected using the enzyme-linked immunosorbent assay (ELISA) kit, and the specific procedures were conducted as instructed (Mlbio, Shanghai, China).

### 2.4. Flow Cytometry

Colon tissues were removed from the mice and then digested with 250 ng/mL of collagenase type I (Sigma-Aldrich) and 20 mg/mL of DNaseI (Sigma-Aldrich) for 2 h at 37°C. After being repeatedly washed, cells were passed through a wire mesh screen and supernatants were centrifuged at 1500 rpm for 10 min. The cell suspension was collected and resuspended with PBS, and the single-cell suspension was incubated with antibodies at 4°C for 30 min. The antibodies were as follows: CD8, B220, CD11b, Gr-1, CD11c, and F4/80. The antibodies were purchased from Biolegend (San Diego, CA), BD Biosciences (San Jose, CA), and eBioscience (San Diego, CA). After washing with PBS, the cell suspension was collected by centrifugation. In intracellular molecular staining, the cells stained with a marker were perforated with Triton 100 for 5 min, washed, and stained with the intracellular molecular antibody (FOXP3) for 30 min. The cells were washed and centrifuged and subsequently detected by flow cytometry.

### 2.5. Immunofluorescence Staining

The mice were divided into the model (DSS), experimental (DSS + DGBX), and control (normal saline) groups, with each group consisting of 6 mice. On the day before each specific time point (days 3, 5, and 9), bromodeoxyuridine (BrdU) with a concentration of 1 mg/mouse was injected into the mice intraperitoneally. After 24 h, the colonic tissue of the mice was washed with PBS and placed in an embedding device with an optimal cutting temperature (OCT) compound. The embedded tissue was directly frozen in a refrigerator at −80°C. The tissue was sliced into sections measuring 10 *μ*m, fixed with 4% paraformaldehyde for 10 min, and sealed with an immunostaining blocking solution for 60 min. BrdU dyeing was conducted as instructed (Sigma-Aldrich, St. Louis, USA). The BrdU antibody was added based on the concentration of 1 : 100, incubated overnight at 4°C, and washed with PBS three times for 5 min each time. Subsequently, the Cy3-conjugated antibody was incubated at 37°C for 30 min. After PBS cleaning, staining with a nuclear dye DAPI (Beyotime Biotechnology, Shanghai, China) for 10 min was conducted. Fluorescence was observed and photographed using an Olympus confocal microscope (BX53 microscope, Olympus, Japan).

### 2.6. Histological Analysis

The distal-colon tissue (1 cm thick) of the mice was fixed with 4% paraformaldehyde, sliced into 2 *μ*m sections after paraffin embedding, and stained with hematoxylin and eosin (Beyotime Biotechnology, Shanghai, China) for 3 min. The microscope (BX53, Olympus, Tokyo, Japan) was used to observe and take pictures. The samples for histological scoring were evaluated using the reference literature [8–10] and graded based on crypt architecture (normal as 0 and severe crypt distortion as 3), the degree of inflammatory cell infiltration (normal as 0 and dense inflammatory infiltrate as 3), muscle thickening (normal as 0 and marked muscle thickening as 3), crypt abscess (absent as 0 and present as 1), and goblet cell depletion (absent as 0 and present as 1). Each mouse was scored as the sum of the aforementioned scores.

### 2.7. Statistical Analysis

All experiments were conducted independently more than three times. The data are presented as shown in the statistical table. Unpaired Student's *t*-test or two-way ANOVA test was used to compare the difference between groups. Statistical significance was defined as *p* < 0.05. All data were analyzed using the software GraphPad Prism 7.0.

## 3. Results

### 3.1. DGBX Delayed the Onset of IBD

**Figure 1:**
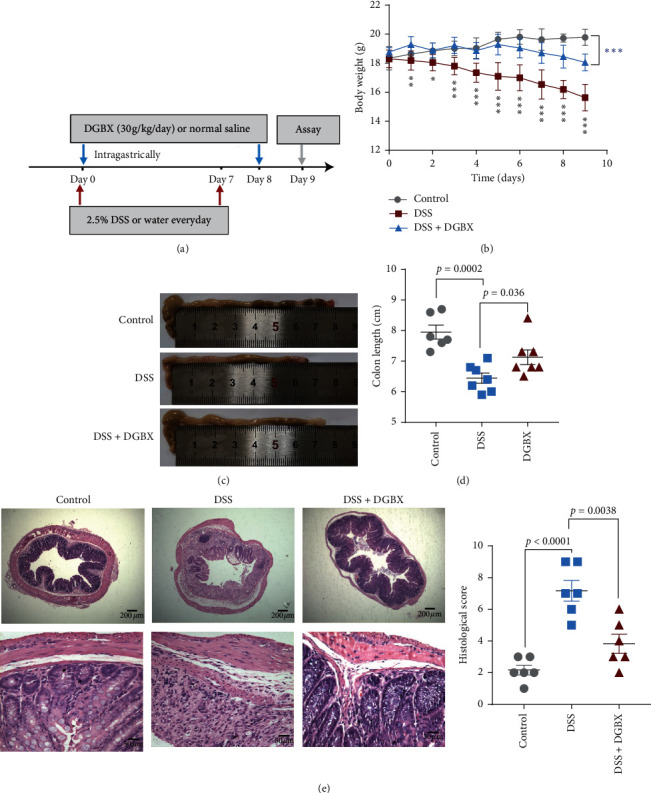
DGBX can effectively prevent the occurrence of DSS-induced IBD. (a) Schematic of DSS modeling and drug administration, with 2.5% DSS treatment for 7 d with ordinary water as the normal control. The DGBX decoction or normal saline was fed intragastrically at 0.4 ml/day on day 0 for 8 d; the mice were killed and analyzed on day 9. Changes in the bodyweight (b) and colon length of the mice (c, d) in the control group (*n* = 6), DSS group (*n* = 7), and DSS + DGBX group (*n* = 7). (e) Hematoxylin and eosin staining of the distal colon sections and corresponding histological scores 9 days after DSS induction (*n* = 6 per group). The black asterisk denotes the comparison between the DSS + DGBX group and the DSS group, and the blue asterisk denotes the comparison between the DSS group and the control group. *∗p* < 0.05,^*∗∗*^*p* < 0.01,  and ^*∗∗∗*^*p* < 0.001. Data are representative of three independent experiments, analyzed using the two-tailed unpaired *t*-test. Bar graphs denote the mean ± standard error of the mean (s.e.m.).

Mice were divided into three groups: the group that was fed with drinking water and given normal saline intragastrically, the group that was fed with DSS water to induce enteritis and given DGBX intragastrically (Figure 1(a)), and the group that was given normal saline intragastrically during DSS induction. Daily changes in weight in mice were recorded. The results indicated that, in the DSS group, the body weight of the mice decreased continuously, indicating that the mouse model of DSS-induced IBD was successfully constructed. Simultaneously, the weight loss of the mice in the DGBX treatment group was significantly alleviated. Even on day 6 of DSS induction, the body weight of the mice was the same as that of the drinking water group, and the body weight decreased from day 7; however, the degree of weight loss was significantly lower than that in the normal saline control group (Figure 1(b)). In addition, the shortening of the colon length is typically an alternative macroscopic indicator of colon injury. We killed the mice on day 9 and measured the length of the colon in each group. The results showed that the colon length was significantly lower in the DSS group than in the drinking water group, and the colon length was significantly longer in the DGBX group than in the DSS group (Figures 3(c) and 3(d)). We then used HE staining to evaluate the severity of colitis. Histological scoring was based on crypt architecture as well as the degree of inflammatory cell infiltration and muscle thickening. The results showed that the DSS group exhibited severe pathological changes, such as the extensive destruction of the crypt structure and a large number of inflammatory cell infiltrates. By contrast, after treatment with DGBX, the mice showed significantly reduced pathological changes, a well-preserved mucosal structure, limited inflammatory reaction, and a slight crypt loss, leading to a decrease in histological scores (Figure 3(e) and Table 1). The aforementioned result showed that DGBX therapy exerted a protective effect on the model of DSS-induced IBD.

### 3.2. DGBX Can Effectively Treat IBD

**Figure 2:**
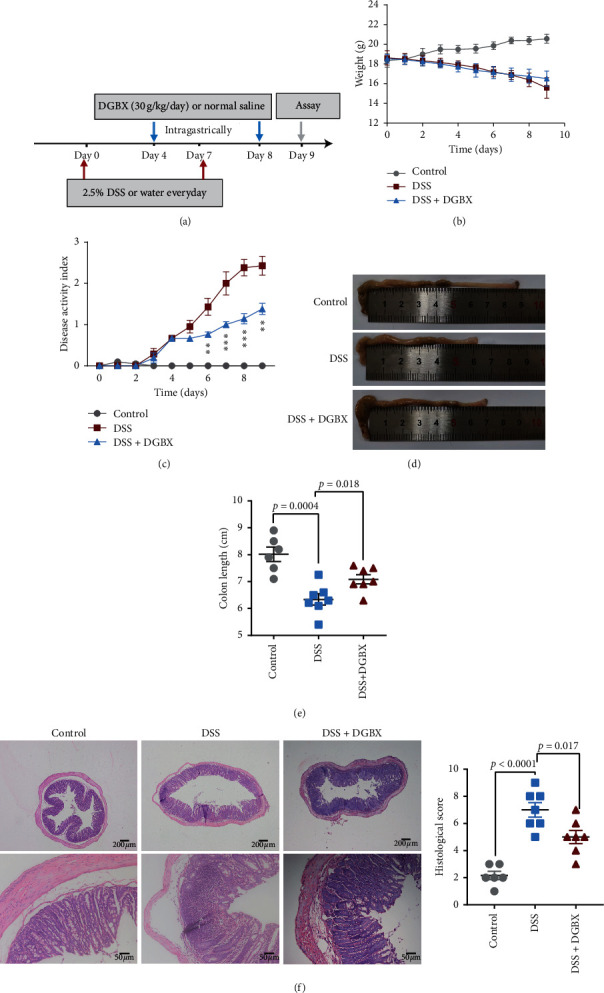
DGBX can effectively treat IBD. (a) Schematic of DSS modeling and drug administration and 2.5% DSS treatment for 7 d with ordinary water as the normal control. The DGBX decoction or normal saline was fed intragastrically at 0.4 mL/on day 4; the mice were killed and analyzed on day 9. Changes in the bodyweight (b), disease activity index (c), and colon length of the mice (d, e) are shown. Hematoxylin and eosin staining of the distal colon sections and corresponding histological scores 9 d after DSS induction. Control group (*n* = 6), DSS group (*n* = 7), and DSS + DGBX group (*n* = 7). (f) Data are representative of three independent experiments and were analyzed using the two-tailed unpaired *t*-test. Bar graphs denote the mean ± standard error of the mean (s.e.m.).

We observed the preventive effect of DGBX on experimental IBD. To elucidate the therapeutic effect of DGBX on IBD, DGBX was given on day 4 (Figure 2(a)). The colitis symptoms of more than half of the mice (such as soft or loose stool, diarrhea, bleeding of visual granules, severe bloody stool, or dark stool), as well as the progress of the disease, were monitored. Although DGBX treatment could not effectively control the continuous weight loss of the mice (Figure 2(b)), it significantly improved the clinical symptoms, such as bloody stool and diarrhea (Figure 2(c)). In addition, after DGBX treatment, colon shortening was significantly improved (Figures 2(d) and 2(e)), and the degree of inflammatory cell infiltration in situ and the destruction of the crypt structure were effectively alleviated (Figure 2(f), Table 2). DGBX treatment could not effectively control the weight loss of the IBD model mice, which could be attributable to the fact that the manifestation of weight loss is usually later than the improvement of intestinal mucosal injury, the effective control of inflammatory cell infiltration, and the remission of clinical symptoms. In summary, the aforementioned results show that DGBX not only has a preventive effect on the experimental IBD but also exerts a significant therapeutic effect on IBD.

### 3.3. DGBX Improved Inflammation in IBD

**Figure 3:**
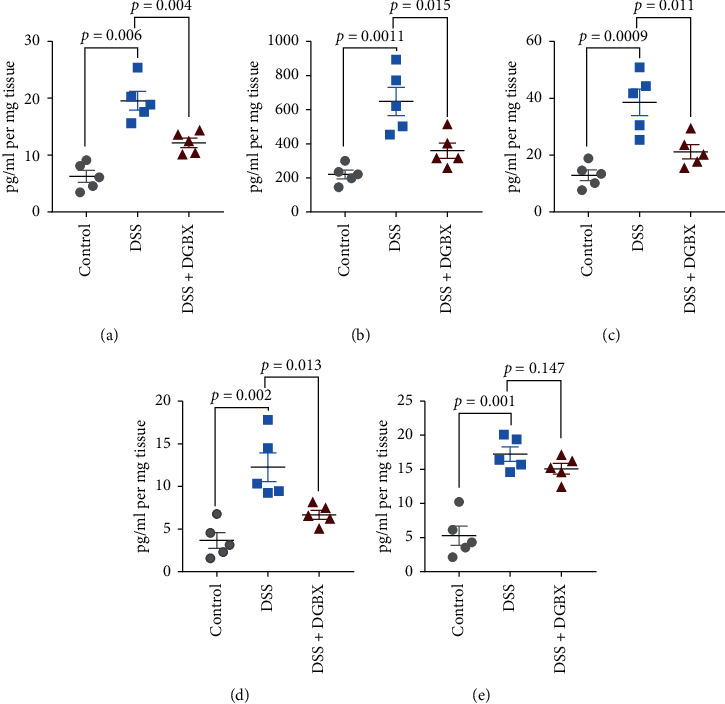
DGBX improves the inflammation of IBD. Analysis of inflammatory cytokines from the distal-colon tissue 9 d after DSS induction (*n* = 6 per group). Data are representative of three independent experiments, analyzed using the two-tailed unpaired *t*-test. Bar graphs denote the mean ± s.e.m. (a) IL-1*β*. (b) TNF-*α*. (c) IL-6. (d) IL-10. (e) IL-17A.

The immune response of intestinal mucosal imbalance is the core link in the occurrence and development of IBD. Production of a large number of inflammatory factors is the key factor influencing the occurrence of IBD [6]. Thus, after the mice were sacrificed on day 9, we further detected the related inflammatory factors in the colon of the model mice to evaluate the inflammation in the local microenvironment of the intestinal mucosa in each group. Colon tissue was collected and homogenized, and the supernatant was obtained after centrifugation. We evaluated the production of proinflammatory cytokines by ELISA. We measured the concentrations of IL-1*β*, TNF-*α*, IL-6, IL-10, and IL-17A in the colon tissue. The results showed a notable distribution of a high concentration of inflammatory factors in mouse colon tissue in the DSS model group. Treatment with DGBX also significantly reduced the production of proinflammatory cytokines, including IL-6 and IL-1*β*, TNF-*α*, and IL-10; by contrast, the IL-17A levels were not affected (Figure 3). All results showed that DGBX can significantly reduce the inflammatory reaction in the colonic tissue of the DSS-induced colitis model.

### 3.4. Effect of DGBX Decoction on the Infiltration of Immune Cell Populations

**Figure 4:**
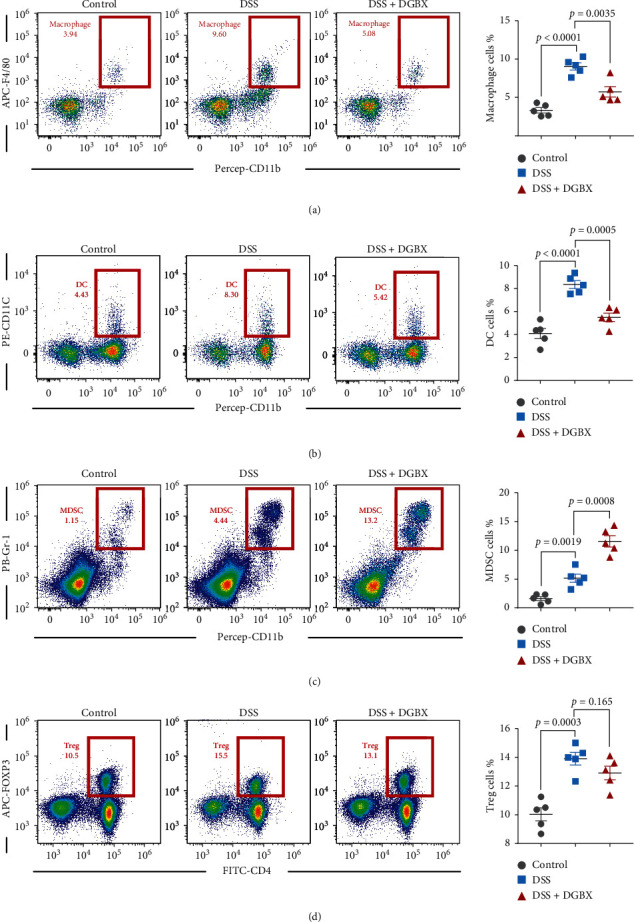
Effect of DGBX decoction treatment on the infiltration of immune cell populations. Analysis of macrophages, dendritic cells, bone marrow-derived suppressor cells, and Tregs from colonic tissue 9 d after DSS induction (*n* = 6 per group). Dot plots are gated on CD45^+^ cells. The numbers adjacent to the outlined areas indicate the percentage of the gated population in each group. Data are representative of three independent experiments, analyzed using the two-tailed unpaired *t*-test. Bar graphs denote the mean ± s.e.m.

After confirming the effect of DGBX on numerous inflammatory factors in the colon, we further assessed the effect of DGBX on the related immune cells in the colon of the model. Previous studies have suggested that macrophages, DCs, MDSC, Tregs, and other cells may be involved in the pathogenesis of DSS-induced colitis, and these cells are highly associated with innate immunity and adaptive immunity [7, 8, 11]. The results of our study suggest that, during the onset of colitis, the proportion of macrophages, DCs, MDSCs cells, and Treg cells increased to varying degrees. The proportion of macrophages, DCs, and other immune cells decreased, whereas the proportion of Treg cells did not significantly change after treatment with DGBX. MDSCs were reported to significantly increase in the tissues of patients with colitis and were confirmed to exert a protective effect on colitis [25]. Some studies found that transfusion of MDSCs exhibited a therapeutic effect on the DSS-induced IBD model. Therefore, MDSCs effectively alleviate the progression of colitis. The results of our study also confirmed the increase in MDSCs in the IBD model mice and verified that the Danggui Buxue decoction could further increase the proportion of MDSCs (Figure 4). These results suggest that DGBX may protect IBD by reducing key cell populations that lead to colonic inflammation, such as macrophages and DCs, as well as increasing cell populations that inhibit colonic inflammation, such as MDSCs.

### 3.5. DGBX Enhanced the Proliferation of  Crypt Epithelial Cells in DSS-Induced Colitis

**Figure 5:**
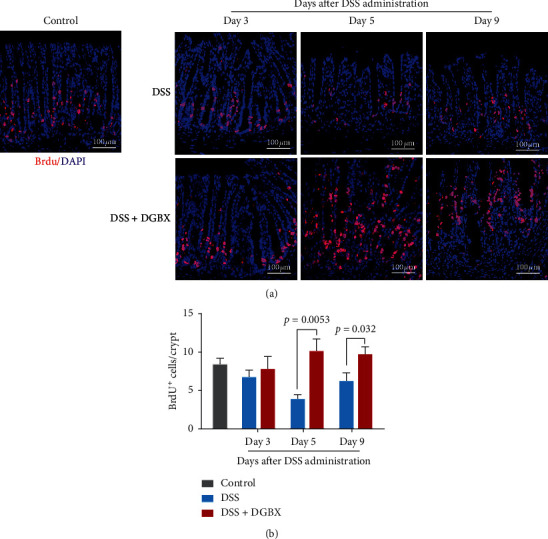
DGBX decoction enhanced the proliferation of crypt epithelial cells in DSS-induced colitis. (a) BrdU staining of distal-colon tissues from mice on days 3, 5, and 9 since DSS administration (*n* = 6 per group). (b) Proportion of BrdU^+^ colonic epithelial cells in colon crypt cells. Data are representative of three independent experiments, analyzed using the two-tailed unpaired *t*-test. Bar graphs denote the mean ± s.e.m.

In the pathological evolution of IBD, intestinal mucosal barrier injury/reconstruction is another key factor for determining the severity and prognosis of IBD. Complete remission of IBD not only requires the regression of inflammation but also depends on the repair of the intestinal mucosal barrier [12, 13]. Continuous proliferation of intestinal cells to replenish the damaged intestinal epithelial cells is the key to the reconstruction of the intestinal epithelial mucosal barrier. To further clarify the mechanism underlying the protective effect of DGBX on IBD, we performed immunofluorescence staining to detect the mouse colonic epithelial cells after intraperitoneal injection of BrdU at different time points induced by DSS. We then evaluated the degree of proliferation by the degree of BrdU incorporation. The results indicated that, from day 3 of IBD induction, the proliferative ability of the intestinal cells was significantly damaged by DSS induction and slowly restored on day 9. However, the degree of cell proliferation of the group treated with DGBX was significantly higher than that of the control group treated with DSS alone; moreover, the proliferative ability of the DGBX group continuously increased until day 9. The intestinal epithelial cells in the DGBX-treated group were repaired more quickly (Figures 5(a) and 5(b)). These results suggest that DGBX treatment not only changes the cell composition of the intestinal local inflammatory microenvironment but also protects the DSS-induced IBD model by enhancing the repairability of intestinal epithelial cells.

## 4. Discussion

IBD is an autoimmune disease characterized by chronic inflammation of the intestinal tract and has become one of the major diseases of the digestive system [26]. Although the pathogenesis of IBD has yet to be clarified, the imbalance of intestinal mucosal immunity and the destruction of intestinal epithelial barrier function play an important role in IBD [27]. The immune response of intestinal mucosal imbalance is the core link in the occurrence and development of IBD. Studies have shown that inflammatory cells such as macrophages and DCs are abnormally activated in the pathogenesis of IBD, and immunosuppressive cells, such as MDSCs, have quantitative or functional defects [7, 11]. Therefore, the restoration of intestinal immune homeostasis is regarded as crucial to the prevention and treatment of IBD. Intestinal mucosal barrier injury/reconstruction is another key factor in determining the severity and outcome of IBD. Complete remission of IBD not only requires the regression of inflammation but also depends on the repair of the intestinal mucosal barrier. Previous studies have confirmed that the differentiation of intestinal stem cells into goblet cells is blocked, leading to insufficient mucin secretion, resulting in the loss of mucous membrane between the intestinal lumen and intestinal epithelium, loss of the intestinal epithelial barrier, and entry of bacteria into intestinal epithelial tissue to cause inflammation, inducing IBD [28]. Therefore, to enhance the proliferative ability of intestinal cells to supplement damaged intestinal epithelial cells, reconstruction of the intestinal epithelial mucosal barrier is another important means to alleviate or treat IBD [12]. Therefore, the alleviation of inflammation in IBD and the reconstruction of the intestinal mucosal barrier are important to determine the outcome of IBD.

DGBX, which is widely prescribed, plays an important role in the treatment of anemia, inflammation, and cardiovascular diseases [29–31]. DGBX reportedly contains astragaloside IV, ferulic acid, and other ingredients, which can regulate immunity and repair epithelial cells [32–34]. In this experiment, DGBX was used to interfere with an IBD mouse model. The results showed that it exerted a protective effect on the IBD model. This effect could significantly alleviate the rate of weight loss, improve colon shortening, and maintain the integrity of the intestinal barrier. DGBX can not only significantly improve the immune response of intestinal mucosal imbalance (such as reducing macrophages and DCs that aggravate the symptoms of IBD) but also upregulate MDSC, which can improve the symptoms of colitis, to control the IBD immune response. DGBX treatment can also increase the proliferative ability of colonic epithelial cells in the IBD colon, allowing the quick repair and replacement of the damaged intestinal epithelial cells, as well as realizing the rapid reconstruction of the intestinal mucosal barrier. On the one hand, it can prevent bacteria from further entering intestinal tissue, thereby preventing a broader immune response; on the other hand, it can prevent a large number of inflammatory factors caused by the imbalance of intestinal mucosal immunity to further damage and destroy intestinal epithelial cells. The results obtained in the current study indicate that DGBX significantly reduced the abnormally increased cytokines, such as IL-1B, TNF-a, IL-6, and IL-10, in the colon of the model mice and significantly alleviated the intestinal inflammatory reaction of the DSS-induced IBD model. This information suggests that DGBX exerts a potential therapeutic effect on IBD. Therefore, the protective effect of DGBX on IBD depends not only on its regulation of the immune system to eliminate the abnormal inflammatory reaction of the intestinal mucosa; the effect also relies on the ability of the intestinal epithelial cells to repair the intestinal mucosal barrier.

## 5. Conclusion

In conclusion, DGBX can simultaneously improve the two main pathogenic mechanisms of IBD: abnormal inflammation and damage to the intestinal mucosal barrier. This study defines the preventive role, also suggests a therapeutic role of DGBX in the DSS-induced IBD model, and improves the understanding of the scope of application of DGBX. Further research can help promote the clinical application prospect of DGBX in IBD.

## Figures and Tables

**Table 1:** The scores of the histological analysis for Figure 1(e).

	Control group	DSS group	DGBX group
Crypt architecture	0, 0, 0, 1, 1, 1	3, 2, 2, 3, 2, 3	1, 1, 1, 1, 2, 1
Degree of inflammatory cell infiltration	1, 1, 2, 1, 1, 0	1, 2, 2, 2, 2, 2	1, 1, 2, 2, 2, 1
Muscle thickening	0, 1, 1, 1, 0, 1	1, 2, 1, 3, 1, 3	1, 1, 1, 2, 1, 0
Crypt abscess	0, 0, 0, 0, 0, 0	1, 1, 0, 1, 0, 1	0, 0, 0, 1, 0, 0
Goblet cell depletion	0, 0, 0, 0, 0, 0	1, 0, 1, 0, 0, 0	0, 0, 0, 0, 0, 0
Total (histological score)	1, 2, 3, 3, 2, 2	7, 7, 6, 9, 5, 9	3, 3, 4, 6, 5, 2

**Table 2:** The scores of the histological analysis for Figure 2(f).

	Control group	DSS group	DGBX group
Crypt architecture	1, 0, 1, 1, 0, 1	2, 3, 3, 3, 2, 2, 3	1, 0, 1, 1, 1, 1, 1
Degree of inflammatory cell infiltration	0, 2, 1, 1, 1, 1	2, 1, 2, 1, 3, 2, 1	2, 2, 2, 2, 2, 2, 2
Muscle thickening	0, 0, 0, 1, 1, 1	1, 1, 2, 2, 3, 1, 1	1, 1, 2, 2, 2, 1, 2
Crypt abscess	0, 0, 0, 0, 0, 0	0, 0, 1, 1, 0, 1, 0	0, 0, 0, 1, 0, 1, 1
Goblet cell depletion	0, 0, 0, 0, 0, 0	1, 1, 1, 1, 0, 1, 0	0, 0, 0, 0, 0, 0, 1
Total (histological score)	1, 2, 2, 3, 2, 3	6, 6, 9, 8, 8, 7, 5	4, 3, 5, 6, 5, 5, 7

## Data Availability

The datasets used during the current study are available from the corresponding author upon reasonable request.
